# Perceptions of graduating students from eight medical schools in Vietnam on acquisition of key skills identified by teachers

**DOI:** 10.1186/1472-6920-8-5

**Published:** 2008-01-20

**Authors:** Luu Ngoc Hoat, Nguyen Minh Son, E Pamela Wright

**Affiliations:** 1 Biostatistics and Medical Informatics Department, Faculty of Public Health, Hanoi Medical University, Dong Da, Hanoi, Vietnam; 2 Epidemiology Department, Faculty of Public Health, Hanoi Medical University, Dong Da, Hanoi, Vietnam; 3 Medical Committee Netherlands – Vietnam, Dong Da, Hanoi, Vietnam

## Abstract

**Background:**

The eight main Vietnamese medical schools recently cooperated to produce a book listing the knowledge, attitudes and skills expected of a graduate, including specification of the required level for each skill. The teaching program should ensure that students can reach that level. The objective of this study was to determine the perception of graduating students on whether they had achieved the level set for a selection of clinical and public health skills as a guide for the schools to adjust either the levels or the teaching.

**Methods:**

From all eight schools, 1136 of the 1528 final year students completed questionnaires just before completed all the requirements for graduation, a response rate of 87% overall (ranging from 74–99% per school). They rated their own competence on a scale of 0–5 for 129 skills selected from the 557 skills listed in the book, and reported where they thought they had learned them. The scores that the students gave themselves were then compared to the levels proposed by the teachers for each skill. The proportions of the self-assessed achievement to the levels expected by the teachers, means self-assessed scores and the coefficients of variation were calculated to make comparisons among disciplines, among schools and among learning sites.

**Results:**

Most students felt they had learned most of the skills for key clinical departments to the required level; this varied little among the schools. Self-assessed skill acquisition in public health and minor clinical disciplines was lower and varied more. Sites outside the classroom were especially important for learning skills. The results revealed key similarities and differences between the teachers and the students in their perception about what could be learned and where

**Conclusion:**

Revising a curriculum for medical schools demands inputs from all stakeholders. Graduating class students can provide valuable feedback on what they have learned in the existing system. Learning objectives should always be checked with students who have followed their study under existing teaching conditions. The information from the graduates helped to identify potential problem areas where either the objectives or the teaching need adjustment.

## Background

University students should know what they are expected to learn during their years of study. In the Netherlands, for example, the learning objectives for medical students were formulated in the Blueprint Book, first published in 1994 and updated in 2001 [1,2]. In the UK, a review of the competencies needed by the modern medical graduate was published in the document called 'Tomorrow's Doctors' in 1993 and updated in 2003 [3,4]. In Vietnam, the training path is different from in these two countries; when students complete their 6^th ^year successfully and graduate, they go directly into practice in a hospital or health centre. The medical curriculum had always been set using a broad framework without specific learning objectives. From 1999 to 2005, a consortium of the eight main medical schools throughout the country (shown in Table 1, in which schools were coded using capital letters from A to H) had support from the Netherlands Government to strengthen their teaching and to make it more community-oriented. The process started with identification of the knowledge, attitudes and skills (KAS) that every general medical graduate from every medical school in Vietnam should have when they leave the school and start to work in the health service. In the first step, approximately 1000 teachers in the eight schools developed the draft KAS book using a participatory and stepwise approach [5]. The draft KAS was then checked against the reality of the working situation after graduation [6] before it was proposed as a basis to revise the curriculum.

**Table 1:** General information about the medical schools involved in the project

**Name of school (coded)**^(1)^	**Year was setup**	**No. of staff (2003)**	**No. of 6**^**th **^**year general medical students (2003)**^(2)^	**Catchment areas**	**Availability of school skill-lab**^(3)^	**No. of field teaching sites**^(4)^
School A	1902	917	359	Whole country	Yes (2004)	5
School B	1947	982	286	Whole country	Yes (1999)	3
School C	1957	523	219	Central & Coast (15 provinces)	Yes (2004)	3
School D	1968	327	240	Northern mountains (12 provinces)	Yes (2005)	5
School E	1968	374	93	Red River Delta (8 provinces)	Yes (1999)	4
School F	1977	100	95	Central Highland (4 provinces)	No	2
School G	1979	325	106	Mekong River Delta (17 provinces)	Yes (1996)	3
School H	1979	203	130	North East & Coast (8 provinces)	No	3

**Total**			**1,528**			

* Note:^(1) ^These schools have produced about 1,500 – 2,000 new general medical doctors each year, who will serve in catchment areas covering from 80 – 90% population in Vietnam. Three medical schools were not involved are military or provincial schools.

^(2) ^Numbers of the 6^th ^year general medical students did not correspond with numbers of staff, since each school were assigned to focus on different kind of students.

^(3) ^Two schools have no skill-lab at school level but there are several small skill-labs at department level and only schools had skill lab before 2003 could contribute for training this study cohort.

^(4) ^All of FT sites are districts in rural or suburban areas, in which there are 15–30 communes with one commune health center in each commune to serve from 3,000 – 10,000 inhabitants.

In the KAS book, the skills are listed with a required level of achievement, either level 1 (can do, but need supervision), level 2, (can do without supervision but not confidently) or level 3 (can do confidently). As the review process progressed, it was recognized that in Vietnamese medical schools, too much time was spent on teaching theory in the classroom and not enough on teaching skills in various settings where students can practice them. The question therefore arose as to whether the medical schools were in fact teaching or would be able to teach the skills to the levels that the teaching staff had proposed as requirements in the draft book. This study was designed to find out from students about to graduate from all eight schools whether they thought they had learned the skills to the level expected by the teachers.

Skills can be learned in a variety of environments. In Vietnamese medical schools, the classroom and the hospital are the main training locations, with the community for a few topics; only a few schools have established skills laboratories (skill-lab). Responses of the students about where they thought they had learned the skills and comparing that to the expected and achieved skill levels can provide evidence to convince the teachers of the importance of the teaching outside the classroom.

This study was therefore designed to document the perception of the students about to graduate from the eight medical schools to help answer the above questions, as a part of the process of finalizing the KAS book that will serve as a frame of reference for the teaching in all the schools.

## Methods

### Study design

The data were collected using a cross-sectional survey among final year students just before they completed all the requirements for graduation. Development of the data collection tools, data entry and analysis was led by Hanoi Medical University, with contributions from each of the others. The study protocol was reviewed and approved by the Scientific Committees in each of the eight medical schools, which are responsible for both technical and ethical aspects of research done by the staff and in the schools. All eight medical schools collected their data using the same methods and tools.

### Study participants

All sixth-year general medical students in 2004 in the eight medical schools listed above were invited to participate in the study. They were called for a class meeting providing them with information about procedures to be completed before they left the schools and the survey was conducted at the end of that meeting. Those who agreed to join (1336 of the 1528) gave verbal consent to the purpose of the study and use of the data. The respondents represented more than 85% of the doctors graduating from these eight schools that year in Vietnam. For six of the schools, participation was between 86 and 99%; only in School A (70%) and School E (74%) was the response lower. Students who did not participate mostly had hospital duty or other activities that prevented them from joining the study; in the latter two schools, the survey was administered late in the day, which may explain the lower proportion of students who joined the meeting and completed the questionnaire, since they have to go to hospitals for evening duty.

### Data collection tool

The 5-page questionnaire was designed as a table so that it could be completed quickly, as shown in Annex (see Additional file 1).

The KAS book specifies one of three levels for each skill, but in this survey, we asked the students to grade their skill on a scale of five. We did this because not all schools taught all skills included in the KAS book and others were only demonstrated, so we added two lower levels (M0, M1) to provide a choice when students may have had no opportunity to practice that skill (not learned at all, or only observed). To facilitate the comparison between the skill levels that the students thought they had achieved and the expected skill levels set by the teachers in the KAS book, we defined skill levels M.2, M.3 and M.4 in the questionnaire as equal to skill levels 1, 2, and 3 in the KAS book, respectively. Responses scored at M0 and M1 were therefore always classified as being below the expected level drafted by the teachers.

The KAS book includes in total 557 specific skills that the teachers considered necessary to deal with the 274 topics in the book. It was not feasible to include all 557 specific skills in the survey. The skills set at level 1 require less time and attention from teachers and students and there was less controversy about their being included in the book. The level 2 and 3 skills need more time in the curriculum and more attention to facilities and methods for teaching them. We therefore to focus on the 290 skills set at level 2 and 13 at level 3. These were still too many for a survey among the students, so we decided to select 40% of these skills using a random numbers table. This proportion was based on the experience from the KAS survey among doctors who had already graduated and were working [6]. Finally we selected 116 skills set at level 2 (can do without supervision but not confidently), and 5 skills set at level 3 (can do confidently). The number of skills level 2 on the questionnaire was slightly higher (124) because some of the selected skills contained sub-skills in the KAS book which were listed separately in the questionnaire.

### Data collection

Experienced researchers from the project teams in each school collected the data after the questionnaire was tested and reviewed with them. Shortly, after the 6^th ^year students had the final theory examinations but in the period of hospital practice assessment, all the students were invited to a meeting where the purpose of the study was explained and the students were asked to complete the questionnaire. The students filled in the forms immediately, under the supervision of the researchers, who collected and checked the questionnaires for completeness. The data were sent to Hanoi Medical University for processing and analysis.

Although the method of self-assessment of skills learned is much less accurate than other methods [7], in this case it was appropriate because the objective was to obtain student feedback on the levels set by the teachers, not to assess precisely the levels achieved.

### Data analysis

In the questionnaires, the students were asked to rate themselves at one of five levels of performance (see data collection tool in Table 1) for each of the selected skills. The mean of the level for each skill reported by the students was compared with the level set by the teachers in the draft KAS book. The means were compared among disciplines and schools to evaluate the success of the teaching/learning process, as perceived by the students. To show the extent of variation in comparison to the mean reported skill achievement among the disciplines, the coefficient of variation (CV) was calculated. That is, if the answers of students regarding a given skill were similar, then the CV would be small, if the responses varied widely, it would be large, regardless of the actual level for that skill.

In analysing and presenting the data, comparisons were made among the different disciplines as established in Vietnamese medical schools. For example, in Vietnam there is a separate Department of Tuberculosis, reflecting the importance of that disease in the health of the nation. For the provision of health care especially at provincial and district levels where most graduates will work, the health services consider that there are four key or core disciplines that must be well represented in every clinical facility: Pediatrics, Obstetrics & Gynecology, Surgery and Internal Medicine. These are allocated more time for both theory and practice in the curriculum than other disciplines.

## Results

### Students' perception on whether they reached the level of skill listed in the KAS book

#### General distribution of responses

The first question was whether the students thought they had learned the required skills to the level expected by the teachers, in general, for all of the skills listed in the questionnaire. The distribution of responses among the students from all the schools taken together is summarized in Table 2. Among the 129 skills studied, the vast majority of the students achieved the expected level for only 16 skills. For another 82 skills (64%), more than half of the students thought that they had reached the level proposed for the KAS book. This comparison revealed which skills many or most students felt they had not learned to do to the level expected by their teachers. For example, only 12% of students reported having acquired the skill of detecting morphine in urine at the level expected by the teachers.

**Table 2:** Distribution of perceived achievement of set skill levels

**% students reporting achievement of the skill level set in the KAS book**	**Frequency**	**Cumulative frequency of skills**	**Percentage**	**Cumulative percentage of skills**
**91–100**	**16**	**16**	**12.4**	**12.4**
**81–90**	**20**	**36**	**15.5**	**27.9**
**71–80**	**15**	**51**	**11.6**	**39.5**
**61–70**	**21**	**72**	**16.3**	**55.8**
**51–60**	**10**	**82**	**7.8**	**63.6**
41–50	19	101	14.7	78.3
31–40	15	116	11.6	89.9
21–30	11	127	8.5	98.4
11–20	2	129	1.6	100.0
0–10	0	129	0.0	100.0

**Total**	**129**		**100.0**	

#### Distribution according to specialization and school

The next question was whether the differences in the frequency of reported skill learning varied among the disciplines represented in the schools as shown in Table 3 below.

**Table 3:** Proportion of students reporting they reached the required level of each skill according to discipline and school

	**Discipline**	**No. of skills**	**Average proportion of students reporting having achieved the level set in the KAS book according to school**								
			
			**A**	**B**	**C**	**D**	**E**	**F**	**G**	**H**	$\bar{X}$
1.	Pediatrics	14	87	85	89	87	95	93	90	87	**89**
2.	Internal Medicine	7	84	86	81	80	96	95	98	81	**88**
3.	Basic	12	81	86	87	84	91	86	92	90	**87**
4.	Surgery	18	82	86	83	81	86	90	90	79	**85**
5.	Dermatology	2	73	79	78	86	96	91	69	77	**81**
6.	ENT	11	78	76	73	80	95	88	74	67	**79**
7.	Nutrition & food safety	2	78	74	62	80	97	75	80	73	**77**
8.	Infectious diseases	1	73	60	84	72	94	88	65	78	**77**
9.	Obstetrics/Gynecology	10	57	91	73	81	85	86	83	54	**76**
10.	Tuberculosis	3	72	78	67	76	88	80	65	70	**75**
11.	Parasitology	2	72	28	61	61	85	76	47	86	**65**
12.	Epidemiology	7	62	57	69	60	55	62	50	55	**63**
13.	Odontostomatology	3	63	37	49	74	82	78	71	53	**63**
14.	Traditional medicine	5	66	60	74	66	51	73	33	71	**62**
15.	Psychiatry	5	61	51	50	64	73	70	50	57	**60**
16.	Ophthalmology	6	46	56	40	64	74	63	59	37	**55**
17.	Health education	6	55	52	37	63	73	48	36	57	**53**
18.	Environmental Health	5	45	27	38	54	86	50	30	49	**47**
19.	Health Management	10	38	43	29	41	67	44	28	41	**41**

	$\bar{X}$		**69**	**70**	**68**	**73**	**85**	**78**	**69**	**68**	**72**

* Note: The third column shows number of skills in each discipline selected for this study, while the last column and last row shows the weighted mean of the proportion of students reporting that they had achieved the required level of skills set by teachers in KAS book, listed according to discipline.

The data in Table 3 show that most of the students in all the schools consistently reported that they had learned the basic skills that all medical doctors need and the skills in the three of the four key clinical departments (Internal Medicine, Surgery and Pediatrics up to the required level in the KAS book (rows 1, 2, 3, 4). For the skills in the other clinical discipline groups, there was more variation among the schools. The proportions of students who thought they had reached the required levels was lower, sometimes dipping to below half. The lowest proportions of reported achievement were for the skills of the three public health disciplines (Health Education, Environmental Health and Health Management); this result was consistent for students from most schools.

The reported achievement of KAS skill levels varied not only among the disciplines but also among the eight schools (Table 3). In School H, for example, students felt they had not reached the set level of skills in several disciplines, with low scores even for key subjects like Obstetrics and Gynaecology. In the larger schools, more students rated themselves as proficient enough in the clinical areas but much less so in the public health fields. Low scores (<60%) were recorded in half or more of the eight schools for Psychiatry, Environmental Health and Health Management. In the smaller schools, such as School E, F and D, most of the students reported achieving most of the skills in all fields to the required level.

### Students' perception of skill achievement

#### Distribution of average skill level achievement according to discipline

**Figure 1:**
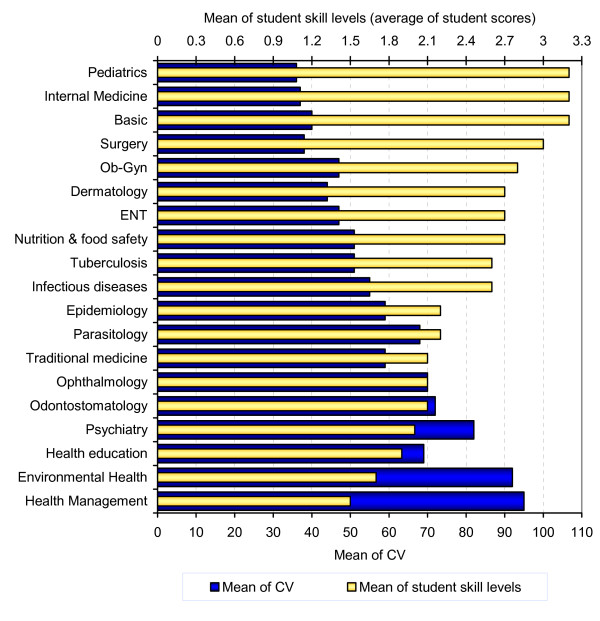
Distribution of means of student skill levels and CV according to discipline.

Figure 1 illustrates the inverse relationship between the mean perceived level of achievement and the variation (CV) in scores among the skills in different disciplines. The highest means scores were in Pediatrics, Internal Medicine and Basic skills, which also had the lowest or very low variation. The mean level of skill achievement was lowest for the topics in health management, while the CV was highest. The difference was clear between the high levels of skill achievement with low variation in most of the clinical departments and the opposite in the public health departments. A few clinical disciplines, such as psychiatry, showed a pattern like public health.

#### Skills with extremely high or low achieving level according to the perception of students

Table 4 shows skills for which more than 90% of the students thought that they had reached the level proposed in the KAS book. The means were high while the CV were very small for those skills. It means that most students rated their learning at a high level for these skills and very few of them rated themselves at a low level. All the skills in Table 4 belong to clinical disciplines where students had more time and opportunity to practice during their six years of study.

**Table 4:** Skills for which students rated their level of skill achievement as high

	**Name of the skill**	**Discipline**	**% met KAS book level**	**Mean of achieving skill level**	**CV**
1.	Identifying the Mac Burney point for diagnosing appendicitis	Surgery	100	3.8	0.6
2.	Chest examination by auscultation and use of the stethoscope.	Basic	97	3.7	0.5
3.	Examining the liver, spleen, and digestive system	Internal medicine	97	3.8	0.5
4.	Examining the upper urinary tract, including kidney.	Internal medicine	96	3.7	0.5
5.	Examination to identify sites of renal calculus formation.	Surgery	95	3.7	0.6
6.	Examination to identify the gall bladder	Surgery	93	3.6	0.7
7.	Examination of the heart, including the recognition of normal and abnormal heart sounds.	Internal medicine	93	3.4	0.6
8.	To identify the pressure point in clinical diagnosis of acute pancreatitis.	Surgery	93	3.6	0.7
9.	To identify symptoms of meningitis.	Pediatrics	92	3.4	0.7
10.	To identify symptoms of acute asthma.	Pediatrics	92	3.6	0.8
11.	To identify pleuritis by auscultation.	Basic	92	3.4	0.8
12.	Taking patient history, including relevant epidemiological factors.	Basic	91	3.5	0.7
13.	Interpretation of laboratory tests (hematology and biochemistry) in children.	Pediatrics	91	3.4	0.8
14.	Examination of the neurological system for brain disorders, level of consciousness and cranial nerve function.	Internal medicine	91	3.3	0.7
15.	Heart auscultation in pediatric care and identification of abnormality.	Pediatrics	91	3.3	0.8
16.	Intravenous and intramuscular injection procedures, including blood transfusion.	Basic	91	3.5	0.7

Table 5, conversely, shows skills for which less than 30% of the students believed they had reached the level set in the KAS book. The means of achieving skill level were low, while the CV were very large. It means that the variation in the students' rating of their skill levels was very high for these skills. It is also clear that most of these skills belong to the field of Public Health (7 of the 12 belong to Health Management) or to the clinical disciplines that students had little time and opportunity to study and to practice.

**Table 5:** Skills for which students rated their level of achievement as low

	**Name of the skill**	**Discipline**	**% met KAS book level**	**Mean of achieving skill level**	**CV**
1.	Making annual and weekly working plan of a health facility	Health management	29	1.5	2.4
2.	Implementation of prioritization policies in community	Health management	29	1.5	2.6
3.	Understanding and interpretation of legal requirements in health care.	Health management	27	1.4	2.7
4.	Implementation and managing health care programs at commune level.	Health management	26	1.4	2.5
5.	Washing the eyes following burn injury.	Ophthalmology	26	1.4	2.5
6.	First aid in penetrating eye injury.	Ophthalmology	25	1.5	2.3
7.	Environmental assessment of school classroom (light, air quality, noise level and appropriate furniture)	Environmental Health	25	1.4	2.7
8.	Legal procedures in health care	Health management	25	1.3	2.8
9.	Testing for abnormality of sight	Ophthalmology	25	2.9	1.2
10.	Practical application of health policies	Health management	22	1.3	2.7
11.	Calculate cover rates (availability, accessibility, usability, completeness, effectiveness) and brief evaluation of cover rates in a health care program	Health management	20	1.1	3.1
12.	Detecting morphine in urine	Psychiatry	12	0.6	5.0

#### Distribution of average skill level achievement of students among schools

**Figure 2:**
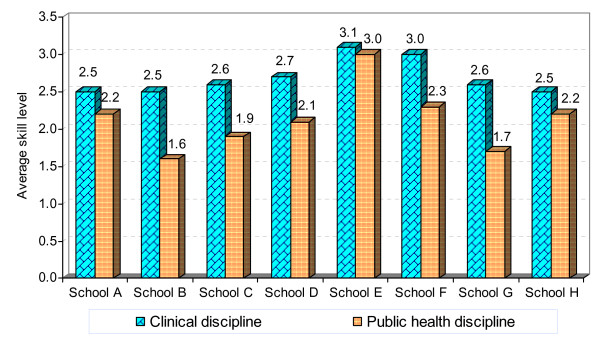
Average of reported skill levels according to school and to clinical or public health discipline.

Eight medical schools joined in this survey. A comparison of the average skill levels reported by the students from the individual schools shown in Figure 2 below reveals differences among the schools.

In all eight schools, there was a higher level of perceived achievement in skills for clinical than for public health disciplines, as shown in Figure 2, but the difference was greater in some schools than others. For example, while the reported achievement of expected skill levels was almost the same for clinical and public health fields in School E, A and H, in School G and B there was a considerable difference between the two. There was also variation among the schools in the average levels that their students felt they had reached in both types of skills by the end of their training. Students in the smallest school (School F) rated themselves higher than those in the bigger and older schools such as School A and B.

### Study sites for learning skills

**Figure 3:**
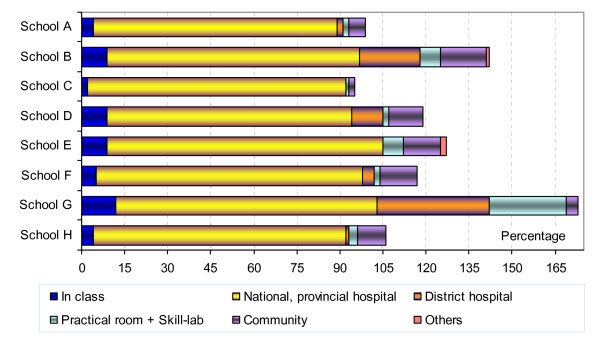
**Study sites for clinical skills in the eight schools**. *Note 1: The data presented in this figure were combined from data of 12 clinical departments: (Internal Medicine, Surgery, Infectious diseases, Obstetrics & Gynecology, Pediatrics, Dermatology, ENT, TB, Traditional Medicine, Ophthalmology, Odontostomatology and Psychiatry. Note 2: One skill may be learned in more than one place, so that totals may be more than 100%.

The students were asked where they thought they had learned the skills. As may be expected, most students learned clinical skills in the central hospitals (Figure 3). For some departments, many students also benefited from training in a district-level hospital, especially for the four key clinical departments (Internal Medicine, Obstetrics and Gynaecology, Surgery, and Paediatrics).

**Figure 4:**
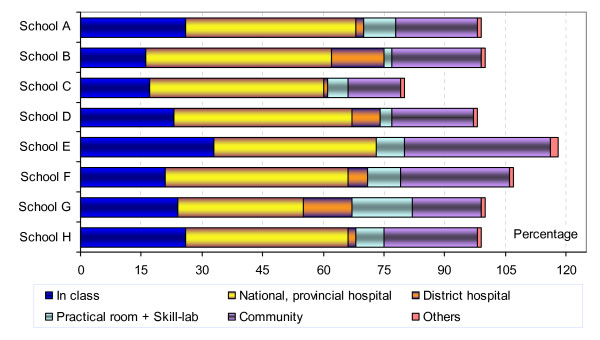
**Study sites for public health skills in the eight schools**. *Note 1: The data presented in this figure were combined from data of five public health departments: Nutrition and Food Safety, Environmental Health, Epidemiology, Health Management, Health Education. Note 2: One skill may be learned in more than one place, so totals may exceed 100%.

For public health departments, many more of the skills were learned in the classroom and the community (Figure 4), although a number of skills, especially in Nutrition, were learned in hospitals as well. The students also reported learning all types of skills from their sessions in the community.

The location 'practical room' included both specialised rooms for laboratory skills as well as facilities for learning pre-clinical and clinical skills (skills-lab). Not all of the schools had a skills-lab for this batch of students to practice; where they did, (in School G, B and E), the students also learned many skills there, especially clinical skills (Figure 3). For the other schools, this location was reported as important only for Parasitology.

## Discussion

The medical schools in Vietnam have been going through a process of formulating the learning objectives for the national curriculum. A draft list of required skills was prepared but there was some question about the capacity of the schools, at present, to train the students to the skill levels defined by the teachers and expected by them. To answer a similar question, Burch et al. assessed 58 graduates from South African medical schools using OSCE and found that their skills were not at the expected level [8]. Self-assessment was used to check the capacity of graduating doctors from Hacetepe University Medical School in Turkey against the requirements of an official job description, and the training was found to be lacking [9].

In this study, feedback from final year students about to graduate made it possible to identify discrepancies between what the teachers expected and what the students thought they had achieved. The process of formulating the revised learning objectives should include the experience of the learners who have gone through the existing training process. What the teachers proposed for the knowledge, skills and attitudes that a graduate should have was not necessarily objective enough, since many teachers proposed levels of skill acquisition for their own field that might not be realistic for the needs of the graduates. The importance of obtaining information from a range of stakeholders to develop an appropriate medical curriculum was emphasized by Snell et al. [10]. Reviewing methods to evaluate a curriculum, they concluded that it was best to make connections between teaching and learning and the expected function of a physician, to lead to the improvement of student learning.

Although self assessment is unreliable as a measure of real achievement of skill learning [7,11] it was not feasible for us to carry out a more objective assessment such as OSCE to evaluate whether the eight medical schools were achieving the level of skill training they aspired to. We therefore asked the graduating general doctors to assess themselves as to the level of expertise they achieved in a number of skills that the teachers consider essential. Because the students had completed all but the very last requirement for graduation and were about to leave the school, we expect that their assessments were as objective as possible; there was little motivation to under- or overestimate them. In any case, we wanted them to report their perception of what they had learned as feedback to the teachers to help finalise the learning objectives and in some cases to reconsider their teaching methods and locations.

The graduates' confidence in their skills varied. Most of the students thought that they had achieved most of the key clinical skills as required by the KAS Book, but were less confident about the public health skills. There was considerable variation among the eight schools in the overall reported level of achievement especially in the public health skills.

The variation in responses can be explained in different ways. The skills in which students gave themselves high scores in most schools were in the subjects that students may have more time for studying, repeat in different years and have more opportunities to practice (internal medicine, surgery, obstetrics and gynaecology, paediatrics). It may also be due to the fact that for these disciplines, the skills and skill levels defined by the teachers in the KAS book are formulated more clearly, more concretely and more suitably for general medical students, so that many students could assess their performance and reach a high level (examples in Table 4). These are the areas that are given importance by the health services and by the medical schools as well, which would probably increase the students' motivation to learn them.

The skills with low mean achievement levels and high CV between schools were often in subjects that are allocated less time in the curriculum or for which there are fewer opportunities to practice, mainly in public health. On the other hand, it may be that the KAS Book levels for these skills were set too high, compared with the capacity and priorities of undergraduate medical students, of teachers and of the teaching program. Another explanation may be that the way of formulating skills belonging to the public health disciplines was not as clear as for the clinical skills, making it more difficult for students to report confidence in performing them (examples in Table 5).

The proportion of students reaching the KAS book skill level in the clinical disciplines was higher than for the public health disciplines and the variation in achievement among schools was less for the clinical skills. Public health teaching is allocated much less time than clinical subjects; many skills may be presented only once in the classroom but never really practiced. Students from schools with better field teaching programs reported higher levels of achievement, perhaps because they had more opportunities to learn the skills in an appropriate setting.

The difference between clinical and public health skills held for all the schools, but there was variation among the schools in several disciplines. The students from the smaller schools tended to report higher achievement levels overall. It is also possible that the skill levels or at least the students' confidence in their skills are in fact higher in the smaller schools, because of more opportunities to practice in hospitals and/or more intensive contact with teachers. In the two biggest schools (School A and B), the undergraduate students have to compete with large numbers of postgraduate students for practice opportunities and for the teachers' time. It is also possible that the higher scores might reflect a tendency for the students in the smaller centres to overestimate their level of skill achievement. These students would have had less exposure to national or regional hospitals where the technical quality of the practice is very high compared to the rural and provincial hospitals farther from the main cities. Students from the larger schools may also have learned to be modest about their level of achievement after their practice in the higher level hospitals.

Whether or not students learn skills thoroughly will depend on their opportunities for practice, and that depends on the locations available in each school. For the curriculum development it was also of interest to know where the students felt they had acquired their skills, because in the detailed curriculum, not only the learning objectives, levels and times but also the recommended location of the teaching is specified. Clinical learning was not only in the higher level hospitals but especially in the smaller schools, the district level hospitals played an important role. The students reported learning all types of skills from their sessions in the community, not only public health. This result may reflect the influence of field teaching programs that were introduced and/or improved during the project, which encouraged teaching of both clinical and public health topics in the field. The concept of learning in a practical room was in the past only applied to teaching laboratory skills, but now most schools want to develop a new style skills laboratory for practice of clinical and other skills. When the school did have a skills laboratory, the students also learned many clinical skills there. School G has the longest-running skills laboratory as well as an established field teaching program that includes the district hospital as a training site. The results from that school reflected the roles of these two sites in skill learning. For other schools where the skill laboratories did not yet exist, the practical room was important only for parasitology.

All of these findings lead us to suggest that the editorial board of the KAS Book and the departments involved should review and reconsider the skills with low means of achieved skill level and high variation. It has been noted in other studies that during self-assessment, students may overestimate their competency [7,11]. If the students in this study overestimated their competency, the need for review and revision of either the requirements or the teaching in low-achievement skills would be even greater. If the skills are indeed necessary at the level set by the teachers, then the training programme will need modification to enable the students to reach the required level. To answer that question, we also carried out a survey among practicing doctors within a few years of graduation. The results are reported in another paper [6] but in general they suggested that most general doctors working in a clinical setting do not need to use the public health skills very often, so that the skills levels should be set lower. Doctors working in public health and needing those skills have usually had additional training in that field.

The study did have some limitations. For example it is difficult to know whether the students who did not complete the survey would have responded differently. Although the overall response rate is high, and the reason for low response rate in two schools was mostly due to hospital duty that was assigned in turn. It is always a limitation to ask people about their own levels of competence, although in this study we did not try to measure competence, but only to collect information about the students' perception of their competence when they were nearly at the end of their study.

International experience of the agreement between defined standards and achieved results came up with comparable recommendations [9]. Even in the UK, a survey of graduates revealed a lack of confidence that all necessary skills had been acquired during training [12]. The process of defining the needed skills in Vietnam was loosely based on the one used in the Netherlands to define the required learning for a graduating doctor [1,2]. In Groningen, the Netherlands, the nationally defined objectives for clinical experiences and skills were compared with the real situation in six clinical clerkships [13]. On average, the clerkships did not fully meet the national objectives, although they did offer clinical experiences that were not mentioned in the formal objectives. The conclusion was that the design of the clerkships should be improved to make them more concordant with national goals. A Danish study asking a similar question about clinical skills of graduates found that the learned skills covered 75% of the intended curriculum and suggested that the curriculum developers should consider the differences in the next round of curriculum development [11]. The field of medical education is still relatively new in Vietnam and many of the processes of reflection and revision of learning objectives and expected learning outcomes are just beginning to develop, whereas in other countries like the UK and the Netherlands, more professional medical and educational institutions have been paying attention to the continual adjustment and improvement of the teaching and learning processes. At this stage in Vietnam, the focus is still very much on the acquisition of good technical skills; a comparison of the KAS book with the outcomes described in other countries [1,4] reveals that we still have to work on the other important aspects of the requirements for medical graduates in Vietnam.

## Conclusion

The results of this study show the importance of consulting all stakeholders in an education process, not least the students who are expected to learn from it. The proportion of students reaching the skill level expected by the teachers varied widely among the skills tested and between the different schools. The reported achievement levels for skills in clinical disciplines were consistently higher than for public health disciplines in this study. For the committee finalising the Vietnamese KAS Book, the study provides evidence that many skills are not being taught, or not learned, to the level proposed in the book. That means that either the level set by the teachers was inappropriate or the teaching programme needs to be adjusted. When the training programmes are revised, attention should be paid to maximising the contributions of field training programmes and other learning sites outside the school. The perceptions of graduating students reveal the extent of their confidence in the ability of the training programme to provide them with the skills they are expected to have to start medical practice. Their reports can contribute to the assessment of the curriculum and help to identify where it needs adjustment to fit with the learning objectives formulated by the teachers.

## List of abbreviations

KAS: book book of knowledge, attitudes and skills expected of a doctor graduating from any Vietnamese medical school; CV: Coefficient of variation; Skill-lab: Skill laboratory.

## Competing interests

The author(s) declare that they have no competing interests.

## Authors' contributions

LNH was the project coordinator; he guided the design, implementation and analysis of the survey, carried out statistical tests and drafted the manuscript. NMS was a team member of the project and joined in the design and data analysis for the survey. EPW was technical adviser to the project, and contributed to the design and planning of the data collection, to data analysis and to drafting the manuscript. All authors read and approved the final manuscript.

## Pre-publication history

The pre-publication history for this paper can be accessed here:



## Supplementary Material

Additional file 1 Questionnaire Questionnaire
