# Supplementary material for: Perceptions of graduating students from eight medical schools in Vietnam on acquisition of key skills identified by teachers
Source: BMC Med Educ. 2008 Jan 20;8:5. doi: 10.1186/1472-6920-8-5 (PMC2248186; doi:10.1186/1472-6920-8-5)
Supplement: Additional file 1 — Questionnaire [file 1472-6920-8-5-S1.doc]

**Annex: Survey to explore perceptions of the 6^th^ year students about to graduate**

*In order to improve the quality of teaching in medical schools, the school is conducting a survey to explore the skills that the students learned during their 6 years and the level appropriate for each skill and the place they think they learned that skill.*

*Notes:*

- *Please read carefully each skill in the table below, identify the existing level that you think that you have achieved for each skill, by checking the appropriate column. (Select the one you think is most appropriate).*
- *Identify where you practiced that skill the most (can select more than one column if appropriate)*

**** Note on skill levels: the level of achievement for each skill is one of five:***

Level 0: not yet learned (M0) Level 1: just observed (never practiced) (M1)

Level 2: can do but need supervision (M2) Level 3: can do but not confidently (M3)

Level 4: can do confidently (M4)

**** Note on sites for learning skill. 1.*** *In class* ***2:*** *Central, provincial hospitals* ***3.*** *District hospitals,*

***4:*** *Skill lab* ***5.*** *Community* ***6.*** *Other places*

| **Code** | **Name of skill** | **The level students believed they had reached for each skill** | | | | | **The place students thought they learned for each skill** | | | | | |
| --- | --- | --- | --- | --- | --- | --- | --- | --- | --- | --- | --- | --- |
|  |  | M0 | M1 | M2 | M3 | M4 | 1 | 2 | 3 | 4 | 5 | 6 |
|  | **1. Basic** |  |  |  |  |  |  |  |  |  |  |  |
|  | Identify pleuritis by auscultation |  |  |  |  |  |  |  |  |  |  |  |
|  | Chest examination by auscultation and use of the stethoscope |  |  |  |  |  |  |  |  |  |  |  |
|  | Intravenous and intramuscular injection, including blood transfusion |  |  |  |  |  |  |  |  |  |  |  |
|  | Taking patient history, including relevant epidemiological factors |  |  |  |  |  |  |  |  |  |  |  |
|  | Physical examination (including pulse, temperature, height/weight) |  |  |  |  |  |  |  |  |  |  |  |
|  | Criteria for ordering X ray and interpret results of X ray |  |  |  |  |  |  |  |  |  |  |  |
|  | Charting fever symptoms |  |  |  |  |  |  |  |  |  |  |  |
|  | Recognize and diagnose myasthenia |  |  |  |  |  |  |  |  |  |  |  |
|  | Identify signs of malnutrition |  |  |  |  |  |  |  |  |  |  |  |
|  | Conduct health education campaign on nutrition |  |  |  |  |  |  |  |  |  |  |  |
|  | Auscultation to detect extrapulmonary fluid in children |  |  |  |  |  |  |  |  |  |  |  |
|  | Identify signs of anemia or jaundice by visual examination |  |  |  |  |  |  |  |  |  |  |  |
|  | **2. Internal medicine** |  |  |  |  |  |  |  |  |  |  |  |
|  | Measure blood sugar using dipstick |  |  |  |  |  |  |  |  |  |  |  |
|  | Assessment of cardiac function, including recognition of normal and abnormal heart sounds |  |  |  |  |  |  |  |  |  |  |  |
|  | Clinical assessment of liver, spleen, and digestive system |  |  |  |  |  |  |  |  |  |  |  |
|  | Emergency cardio pulmonary resuscitation |  |  |  |  |  |  |  |  |  |  |  |
|  | Clinical assessment of upper urinary tract, including kidney |  |  |  |  |  |  |  |  |  |  |  |
|  | Endocrinological assessment of thyroid, pancreas function, etc |  |  |  |  |  |  |  |  |  |  |  |
|  | Neurological assessment for level of consciousness, brain disorders |  |  |  |  |  |  |  |  |  |  |  |
|  | **3. Surgery** |  |  |  |  |  |  |  |  |  |  |  |
|  | Criteria to order and interpret results of stomach X ray with and without barium preparation |  |  |  |  |  |  |  |  |  |  |  |
|  | Criteria to order and interpret results of endoscopy |  |  |  |  |  |  |  |  |  |  |  |
|  | Criteria to order and interpret results of blood tests |  |  |  |  |  |  |  |  |  |  |  |
|  | Identify MacBurney point for diagnosis of appendicitis |  |  |  |  |  |  |  |  |  |  |  |
|  | Procedures to identify sites of renal calculus formation |  |  |  |  |  |  |  |  |  |  |  |
|  | Locate pressure point in clinical diagnosis of acute pancreatitis |  |  |  |  |  |  |  |  |  |  |  |
|  | Locate gall bladder by clinical examination |  |  |  |  |  |  |  |  |  |  |  |
|  | Palpate to identify enlarged spleen, locate bladder |  |  |  |  |  |  |  |  |  |  |  |
|  | Rectal examination to identify Douglas signs |  |  |  |  |  |  |  |  |  |  |  |
|  | Rectal examination to identify enlarged prostate |  |  |  |  |  |  |  |  |  |  |  |
|  | Procedures to identify rectal tumor and distinguish from polyps |  |  |  |  |  |  |  |  |  |  |  |
|  | Identify rectal fistula in children |  |  |  |  |  |  |  |  |  |  |  |
|  | Criteria for circumcision in children |  |  |  |  |  |  |  |  |  |  |  |
|  | Insert gastric tube |  |  |  |  |  |  |  |  |  |  |  |
|  | Identify limb dysfunction due to peripheral nerve damage |  |  |  |  |  |  |  |  |  |  |  |
|  | Clean and dress small wounds |  |  |  |  |  |  |  |  |  |  |  |
|  | Reset dislocated joints |  |  |  |  |  |  |  |  |  |  |  |
|  | **4. Obstetrics/gynecology** |  |  |  |  |  |  |  |  |  |  |  |
|  | Insert speculum |  |  |  |  |  |  |  |  |  |  |  |
|  | Guide how to use condom |  |  |  |  |  |  |  |  |  |  |  |
|  | Examine pregnant women during 3 trimesters |  |  |  |  |  |  |  |  |  |  |  |
|  | Identify stages of dilatation during delivery |  |  |  |  |  |  |  |  |  |  |  |
|  | Identify position of fetus |  |  |  |  |  |  |  |  |  |  |  |
|  | Removal of placenta and check it is intact |  |  |  |  |  |  |  |  |  |  |  |
|  | Artificial rupture of membranes |  |  |  |  |  |  |  |  |  |  |  |
|  | Aspiration of the neonate |  |  |  |  |  |  |  |  |  |  |  |
|  | Prepare Pap smear |  |  |  |  |  |  |  |  |  |  |  |
|  | Counseling for family planning |  |  |  |  |  |  |  |  |  |  |  |
|  | **5. Pediatrics** |  |  |  |  |  |  |  |  |  |  |  |
|  | Identify symptoms of meningitis |  |  |  |  |  |  |  |  |  |  |  |
|  | Refer child to higher level hospital as needed |  |  |  |  |  |  |  |  |  |  |  |
|  | Identify critical symptoms according to IMCI |  |  |  |  |  |  |  |  |  |  |  |
|  | Identify signs and degree of dehydration in children |  |  |  |  |  |  |  |  |  |  |  |
|  | Make and use oral rehydration solution and other rehydrating fluids at home |  |  |  |  |  |  |  |  |  |  |  |
|  | Identify abnormal curvature of the spine (scoliosis) |  |  |  |  |  |  |  |  |  |  |  |
|  | Identify signs and symptoms of acute asthma |  |  |  |  |  |  |  |  |  |  |  |
|  | Identify chest malformation |  |  |  |  |  |  |  |  |  |  |  |
|  | Interpretation of laboratory tests (hematology and biochemistry) in children. |  |  |  |  |  |  |  |  |  |  |  |
|  | Collect stool and urine samples from children and interpret lab test results |  |  |  |  |  |  |  |  |  |  |  |
|  | Recognize three Gallia signs in children |  |  |  |  |  |  |  |  |  |  |  |
|  | Auscultation to detect abdominal fluid in children |  |  |  |  |  |  |  |  |  |  |  |
|  | Recognition of abnormal heart sounds in children |  |  |  |  |  |  |  |  |  |  |  |
|  | Use stethoscope to assess heart valve function |  |  |  |  |  |  |  |  |  |  |  |
|  | Use stethoscope to detect abdominal fluid |  |  |  |  |  |  |  |  |  |  |  |
|  | **6. Ophthalmology** |  |  |  |  |  |  |  |  |  |  |  |
|  | Assess ocular function |  |  |  |  |  |  |  |  |  |  |  |
|  | Evaluate ocular pressure |  |  |  |  |  |  |  |  |  |  |  |
|  | Flush eyes following burn trauma |  |  |  |  |  |  |  |  |  |  |  |
|  | First aid for penetrating eye trauma |  |  |  |  |  |  |  |  |  |  |  |
|  | Bandage eyes as first aid |  |  |  |  |  |  |  |  |  |  |  |
|  | Apply eye drops |  |  |  |  |  |  |  |  |  |  |  |
|  | **7. Odontostomatology** |  |  |  |  |  |  |  |  |  |  |  |
|  | Diagnose acute and chronic oral infections |  |  |  |  |  |  |  |  |  |  |  |
|  | Give first aid to stop bleeding, treat shock or asphyxia, and to prevent infection |  |  |  |  |  |  |  |  |  |  |  |
|  | Organize program to provide dental care for children in community |  |  |  |  |  |  |  |  |  |  |  |
|  | **8. ENT** |  |  |  |  |  |  |  |  |  |  |  |
|  | Diagnose inner ear infection |  |  |  |  |  |  |  |  |  |  |  |
|  | Assess nasal function |  |  |  |  |  |  |  |  |  |  |  |
|  | Diagnose sinusitis |  |  |  |  |  |  |  |  |  |  |  |
|  | Assess tonsils |  |  |  |  |  |  |  |  |  |  |  |
|  | Identify disorders affecting the voice |  |  |  |  |  |  |  |  |  |  |  |
|  | Prescribe appropriately for common ENT diseases |  |  |  |  |  |  |  |  |  |  |  |
|  | Diagnose rhinitis and laryngitis in children |  |  |  |  |  |  |  |  |  |  |  |
|  | Diagnose acute or chronic tonsillitis |  |  |  |  |  |  |  |  |  |  |  |
|  | Detect presence of foreign objects in esophagus |  |  |  |  |  |  |  |  |  |  |  |
|  | Diagnose nasal bleeding |  |  |  |  |  |  |  |  |  |  |  |
|  | Diagnose respiratory problem due to pharyngeal symptoms |  |  |  |  |  |  |  |  |  |  |  |
|  | **9. Infectious diseases** |  |  |  |  |  |  |  |  |  |  |  |
|  | Criteria for isolation |  |  |  |  |  |  |  |  |  |  |  |
|  | **10. Tuberculosis** |  |  |  |  |  |  |  |  |  |  |  |
|  | Recognize symptoms of TB |  |  |  |  |  |  |  |  |  |  |  |
|  | Manage TB patient in community |  |  |  |  |  |  |  |  |  |  |  |
|  | Instruct patient to provide sputum sample |  |  |  |  |  |  |  |  |  |  |  |
|  | **11. Traditional medicine** |  |  |  |  |  |  |  |  |  |  |  |
|  | Insert acupuncture needles |  |  |  |  |  |  |  |  |  |  |  |
|  | Identify acupuncture points to treat eight common symptoms & diseases |  |  |  |  |  |  |  |  |  |  |  |
|  | Manage complications of acupuncture |  |  |  |  |  |  |  |  |  |  |  |
|  | Prescribe traditional medicines to treat eight common symptoms & diseases |  |  |  |  |  |  |  |  |  |  |  |
|  | Apply massage and acupressure |  |  |  |  |  |  |  |  |  |  |  |
|  | **12. Psychiatry** |  |  |  |  |  |  |  |  |  |  |  |
|  | Identify signs & symptoms of common psychiatric disorders |  |  |  |  |  |  |  |  |  |  |  |
|  | Counsel families on supportive care for patients with high risk of suicide |  |  |  |  |  |  |  |  |  |  |  |
|  | Screen urine samples for morphine |  |  |  |  |  |  |  |  |  |  |  |
|  | Detect morphine in the urine |  |  |  |  |  |  |  |  |  |  |  |
|  | Diagnose schizophrenia |  |  |  |  |  |  |  |  |  |  |  |
|  | **13. Parasitology** |  |  |  |  |  |  |  |  |  |  |  |
|  | Stool examination for worm eggs using direct microscopy (Willis, Kato) |  |  |  |  |  |  |  |  |  |  |  |
|  | Prepare thick and thin blood smears |  |  |  |  |  |  |  |  |  |  |  |
|  | **14. Dermatology** |  |  |  |  |  |  |  |  |  |  |  |
|  | Treat common dermatological conditions |  |  |  |  |  |  |  |  |  |  |  |
|  | Appropriate application of salves |  |  |  |  |  |  |  |  |  |  |  |
|  | **15. Environmental health & school hygiene** |  |  |  |  |  |  |  |  |  |  |  |
|  | Environmental assessment of a classroom |  |  |  |  |  |  |  |  |  |  |  |
|  | Instruct teachers how to use growth charts for children |  |  |  |  |  |  |  |  |  |  |  |
|  | Identify sources of pollution in community and in hospitals |  |  |  |  |  |  |  |  |  |  |  |
|  | Prevent and manage occupational fatigue |  |  |  |  |  |  |  |  |  |  |  |
|  | Advise on use and storage of pesticides in community |  |  |  |  |  |  |  |  |  |  |  |
|  | **16.Nutrition & food safety** |  |  |  |  |  |  |  |  |  |  |  |
|  | Community counseling to prevent malnutrition |  |  |  |  |  |  |  |  |  |  |  |
|  | Counseling on appropriate diet for patients |  |  |  |  |  |  |  |  |  |  |  |
|  | **17. Health management** |  |  |  |  |  |  |  |  |  |  |  |
|  | Practical application of health policies |  |  |  |  |  |  |  |  |  |  |  |
|  | Implement priority policies in community |  |  |  |  |  |  |  |  |  |  |  |
|  | Understand and interpret legal requirements |  |  |  |  |  |  |  |  |  |  |  |
|  | Understand legal procedures |  |  |  |  |  |  |  |  |  |  |  |
|  | Implementation and manage health care programs at commune level |  |  |  |  |  |  |  |  |  |  |  |
|  | Report health information from district or commune health center |  |  |  |  |  |  |  |  |  |  |  |
|  | Promote collaboration between community and health staff |  |  |  |  |  |  |  |  |  |  |  |
|  | Prepare annual and monthly work plan of a health facility |  |  |  |  |  |  |  |  |  |  |  |
|  | Apply primary health care principles in implementing a health care program |  |  |  |  |  |  |  |  |  |  |  |
|  | Calculate and assess coverage in a health care program |  |  |  |  |  |  |  |  |  |  |  |
|  | **18. Health education** |  |  |  |  |  |  |  |  |  |  |  |
|  | Identify health education needs for individuals and communities |  |  |  |  |  |  |  |  |  |  |  |
|  | Identify topics appropriate for target groups in health education |  |  |  |  |  |  |  |  |  |  |  |
|  | Plan a community health education campaign on a concrete issue |  |  |  |  |  |  |  |  |  |  |  |
|  | Use appropriate methods for a health education program |  |  |  |  |  |  |  |  |  |  |  |
|  | Select/ use appropriate communication channels in a health education campaign |  |  |  |  |  |  |  |  |  |  |  |
|  | Prepare contents of a health education presentation |  |  |  |  |  |  |  |  |  |  |  |
|  | **19. Epidemiology** |  |  |  |  |  |  |  |  |  |  |  |
|  | Calculate incidence and prevalence rates |  |  |  |  |  |  |  |  |  |  |  |
|  | Select appropriate sample size for a study |  |  |  |  |  |  |  |  |  |  |  |
|  | Select appropriate tools for data collection |  |  |  |  |  |  |  |  |  |  |  |
|  | Indications and contra-indications for administering common vaccines |  |  |  |  |  |  |  |  |  |  |  |
|  | Calculate indicators related to community health |  |  |  |  |  |  |  |  |  |  |  |
|  | Select appropriate sources for data collection |  |  |  |  |  |  |  |  |  |  |  |
|  | Select appropriate content to monitor diseases in the community. |  |  |  |  |  |  |  |  |  |  |  |
